# Exploring the disease burden and quality of life in patients with short bowel syndrome with intestinal failure: Insights from exit interviews in the glepaglutide EASE SBS‐1 phase 3 trial

**DOI:** 10.1002/ncp.70109

**Published:** 2026-03-12

**Authors:** David F. Mercer, Constance Mubekapi‐Musadaidzwa, Bitten Kloster, Mark Berner‐Hansen, Anna Rydén, Palle Bekker Jeppesen

**Affiliations:** ^1^ Department of Surgery University of Nebraska Medical Center, Nebraska Medical Center Omaha Nebraska USA; ^2^ CLINIGMA Copenhagen Denmark; ^3^ Zealand Pharma Copenhagen Denmark; ^4^ Digestive Disease Centre, Bispebjerg University Hospital of Copenhagen Copenhagen Denmark; ^5^ Rigshospitalet, Department of Digestive Diseases, Transplantation and General Surgery Section for Intestinal Failure Copenhagen Denmark

**Keywords:** exit interview, intestinal failure, quality of life, SBS‐IF, short bowel syndrome

## Abstract

**Background:**

A randomized, double‐blind, placebo‐controlled trial was conducted to evaluate efficacy and safety of glepaglutide in patients who have short bowel syndrome with intestinal failure (SBS‐IF). At the end of the trial, exit interviews were conducted to explore participants' experiences and to assess the impact of the disease and treatment during the trial.

**Methods:**

Thirty patients from four countries were interviewed over the phone. Data were collected using a semistructured interview manual, and interviews were recorded and transcribed for analysis.

**Results:**

Patients reported that SBS‐IF negatively impacted their lives before the trial, causing loss of freedom, disrupted sleep, limited physical activity, and pain. During the interviews, patients reported that the treatment improved their well‐being across multiple domains. Seventy‐three percent of the patients receiving glepaglutide (*n* = 16/22) reported positive changes in health‐related quality of life compared with 25% receiving placebo (*n* = 2/8). Twenty‐six patients reported experiencing a reduction in parenteral support (PS) volume. Of these, 21 patients (18 glepaglutide, three placebo) reported a change in overall status, with 94% receiving glepaglutide (*n* = 17/18) and 67% (*n* = 2/3) receiving the placebo finding this change meaningful. Although descriptive, these findings should be interpreted cautiously given the small number of patients.

**Conclusion:**

During exit interviews, patients receiving glepaglutide reported improvements in well‐being across multiple domains, noting meaningful reductions in PS volume and a reduced impact of SBS‐IF on daily life, which was proportionally greater than in those receiving placebo. These findings underscore the patient‐reported positive experiences of glepaglutide and its beneficial effects.

## INTRODUCTION

**Figure 1:**
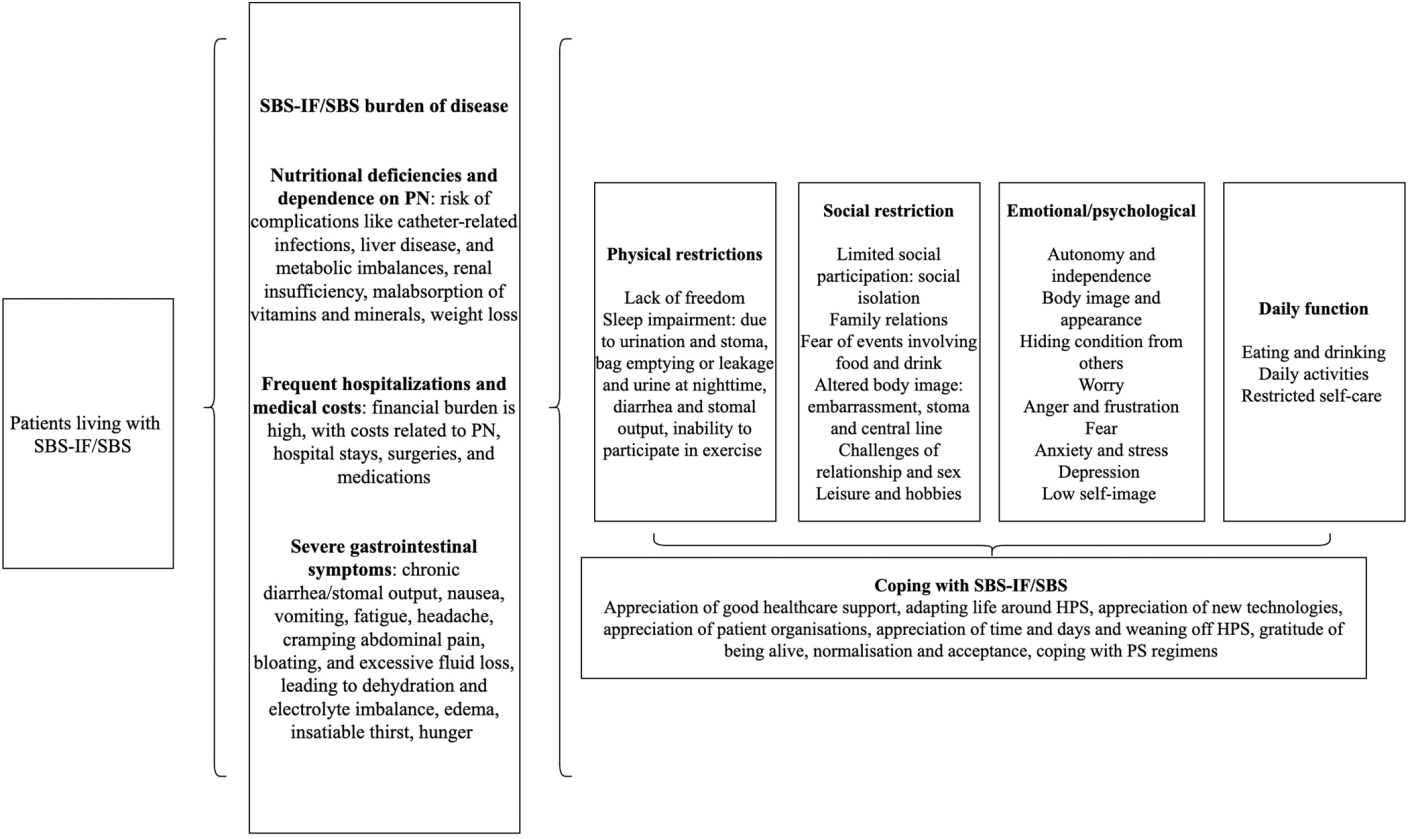
SBS‐IF symptoms and impact model. HPS, home parenteral support; IF, intestinal failure; PN, parenteral nutrition; SBS, short bowel syndrome.

Short bowel syndrome (SBS) is a rare and complex condition defined clinically in adults as having <200 cm of functional small bowel length.^1^, ^2^ It is characterized by a reduced intestinal surface area after resection and dysregulation of the entero‐hormonal secretion leading to impaired intestinal absorptive function.^3^ Given the lack of consistently applied disease criteria and the use of home parenteral support (HPS) as a proxy to estimate the size of the SBS population, prevalence estimates vary from 0.4 to 25 per million people across the United States and selected European countries.^4^, ^5^ A recent publication estimated prevalences of diagnosed SBS with ‐intestinal failure (SBS‐IF) in the general population in 2024 ranged from 1.2 to 27.4 per million in adults and from 0.9 to 16.7 per million in children.^6^ The condition leads to frequent dehydration, electrolyte imbalances, and malnutrition, which manifests as fatigue, headaches, cramping, and edema along with persistent abdominal pain, nausea, vomiting, and insatiable thirst and hunger^7^, ^8^ (Figure 1).

Treatment for SBS is focused on managing dehydration, nutrient loss, debilitating diarrhea, and weight loss.^8^ Parenteral support (PS) varies from intermittent treatments to total PS, administered either at home or in hospitals. Despite being lifesaving, HPS poses several challenges such as admissions due to catheter‐related complications, development of intestinal failure (IF)‐associated liver disease, and disrupted daily routines. These issues lead to hesitancy to engage socially due to body image challenges, fear of events involving food and drink, and the need for toilets. In addition, physical restrictions such as inability to participate in activities, lack of freedom, restrictions in self‐care, and disrupted sleep impact quality of life (QoL).^4^, ^5^, ^9^, ^10^ Emotionally, patients often feel sad, frustrated, and anxious about HPS complications or accidents.^11^ Despite these challenges, patients cope with SBS‐IF by adapting their life around it and finding hope in new technologies and the chance of eventually weaning off HPS. This, coupled with good healthcare and patient organization support, helps them to better cope with SBS.

Recent advancements in SBS drug treatments, particularly with glucagon‐like peptide 2 (GLP‐2) analogues, hold promise for improving outcomes for patients with SBS‐IF.^7^ Glepaglutide is a long‐acting GLP‐2 analogue with subcutaneous administration and an extended half‐life of 88 hours,^12^ enabling significant extension of the dosing interval.^13^ A pivotal randomized, placebo‐controlled trial (Efficacy And Safety Evaluation of Glepaglutide in Treatment of Short Bowel Syndrome [EASE SBS 1]) demonstrated that 24 weeks of twice‐weekly (TW) treatment with 10 mg of glepaglutide significantly reduced or eliminated the need for PS in patients with SBS‐IF.^13^ In alignment with these findings, patients receiving glepaglutide reported significant benefits of the treatment, compared with those receiving placebo (PBO), as evaluated by Patient Global Impression of Change (PGIC) scale, and glepaglutide was found to be safe and well tolerated.^13^

To complement these findings, qualitative exit interviews were conducted with a subgroup of patients from the EASE SBS‐1 trial. The primary aim of the study was to explore the lived experiences of patients with SBS‐IF before and after EASE SBS‐1 treatment. The study sought to understand the physical symptoms, emotional challenges, and daily life restrictions that patients considered most important and impactful to their daily functioning and overall QoL. Whereas quantitative data demonstrated robust evidence of treatment efficacy, qualitative exit interviews offered in‐depth patient‐centered insights into how treatment affects daily functioning and overall well‐being, capturing experiential nuances that quantitative measures may not fully capture. These insights are essential for a comprehensive evaluation of the treatment's effectiveness and for informing future patient‐centered care strategies.

## MATERIAL AND METHODS

### Study design

The exit interviews were a substudy of the EASE SBS‐1 trial. The details on the trial design as well as inclusion and exclusion criteria have been recently published.^13^ The trial involved patients with SBS‐IF with a high and stable need for PS at the time of inclusion. All patients were naïve to glepaglutide and had not been exposed to another GLP‐2 analogue, GLP‐1, or other relevant treatment within 3 months before screening. The vast majority were entirely naïve to GLP‐2 analogues. Eligible patients who completed the trial from sites in the United States, the United Kingdom, Germany (DE), and Denmark (DK); completed the trial within 7 days of the end‐of‐treatment visit; could communicate in their native language; and had given written consent were invited to participate in the exit interviews. Purposive sampling^14^ was used to recruit approximately one‐third of participants from the main clinical trial population for exit interviews. This subset was selected based on predefined eligibility criteria and was representative of the main trial population across key demographic and disease characteristics. The goal was to capture a range of lived experiences and treatment perspectives from a population living with SBS‐IF. Given the heterogeneous nature of SBS, the aim was not to achieve thematic saturation but rather to explore the diversity of participant perspectives. The decision to interview a third of participants balanced the need for variation and representation with practical considerations such as resource availability. Post hoc chi‐square tests showed that the exit interview sample was comparable to the overall study population across key demographic and clinical characteristics, except for country representation, as US participants were overrepresented.

To minimize bias, all interviews were conducted during the double‐blind phase of the study. At the time of the interviews, study coordinators, participants, and interviewers, as well as healthcare professionals at the study sites, remained blinded to the study treatment. This ensured that participants' responses were not influenced by knowledge of whether they had received the investigational treatment or PBO, thereby minimizing bias and preserving the integrity of the qualitative data collected.

Study coordinators at the clinical sites recruited patients and shared their contact details with the external interview team, who then arranged telephone interviews. All interviews were conducted between August 2019 and July 2022 by interviewers who were native speakers of the language used in each respective interview. Each interviewer held a graduate degree in the social sciences and had formal training in qualitative interviewing, with a minimum of 5 years of experience conducting qualitative research. Before conducting interviews, interviewers were briefed on the study's background, objectives, and rationale and received project‐specific training aligned with study expectations. Interviewers had no prior relationship with participants and were assigned interviews without any prior introductions. Interviewers were selected based on their language fluency and expertise in qualitative research. All interviews were conducted via telephone, so participants were unaware of interviewer attributes beyond gender and language. In conducting interviews, researchers followed standardized procedures: confirming participant consent, asking clarifying questions when necessary, avoiding interruptions, maintaining neutrality, refraining from leading questions, and keeping within time limits. Semistructured interviews lasted up to 60 min, were recorded, were later transcribed verbatim for analysis, and, where applicable, were translated to English. No additional compensation was provided to the participants.

The trial adhered to the Declaration of Helsinki, International Conference on Harmonization guidelines, and Good Clinical Practice standards. Institutional review board or independent ethics committee approval was obtained for each center. All participants provided written informed consent. All data collected in the study were maintained as strictly confidential.

### Data collection

The semistructured interview manual was developed following the Food and Drug Administration (FDA) final guidance *Patient‐Focused Drug Development: Collecting Comprehensive and Representative Input. Guidance for Industry, Food and Drug Administration Staff, and Other Stakeholders*.^15^ The questions were generated based on literature review, expert consultations, and patient focus groups, ensuring they were comprehensive and relevant. Both open‐ and closed‐ended questions were used to capture the experiences of the patients with SBS‐IF. Revisions to the interview manual, made after initial interviews, were based on direct communication with the FDA and were focused on clarifying, simplifying, and restructuring questions; adding prompts; and integrating decision trees to enhance clarity and recall. In accordance with the updated manual, only patients who met predefined decision‑tree criteria were asked relevant follow‑up questions. The interview manual addressed meaningful reductions in PS and changes in physical and emotional symptoms. The manual also included a self‐rated 7‐point patient‐reported outcome (PRO) Likert scale. This scale assessed the perceived changes in a patient's condition, with response options ranging from “very much improved” to “very much worsened.” The SBS‐Impact Scale (SBS‐I)—a newly developed, disease‐specific, PRO exploratory questionnaire developed by Zealand Pharma specifically for this study—was used to assess physical and emotional symptoms of SBS‐IF and their impact on daily life. This instrument was created to capture the experiences of patients with SBS‐IF, as no fully validated disease‐specific PRO tool was available at the time. SBS‐I has not undergone formal psychometric validation and has not been used in prior studies. Its use here is intended to provide preliminary insights to guide future research and instrument development in this population. Additionally, demographic and disease characteristics were collected in the main trial to describe the exit interview study population.

### Data analysis

Data were analyzed using both deductive and inductive coding to ensure comprehensive analysis. Deductive coding applied a predefined disease conceptual model derived from a targeted literature review, whereas the inductive coding identified new concepts on the impact of SBS on daily life. Data management was performed using Dedoose (v9.0.46; Sociocultural Research Consultants, LLC). Analysts initially reviewed three transcripts to familiarize themselves with the data. To ensure reliability, 10% of the transcripts were uploaded for training sessions at the Dedoose training center. A senior researcher initially coded the transcripts, and independent analysts (unaware of the original codes) applied their own codes during training. The Cohen kappa coefficient was used to assess interrater reliability, with a score of 1.0 indicating 100% agreement between analysts. The initial tests showed moderate level of agreement, with scores ranging between 0.41 and 0.60. This prompted discussions to clarify code definitions and adjust the codebook, which improved interrater reliability to 0.80–1.00, indicating almost perfect agreement. Researchers reviewed and refined the codes iteratively and identified themes through thematic analysis. Closed‐ended questions were analyzed using Stata/SE 17.0 (StataCorp LLC).

## RESULTS

### Participants

A total of 30 patients consented to the substudy. Of these, 14 received glepaglutide 10 mg once weekly (OW), eight received glepaglutide TW, and eight received PBO. Data from the baseline demographics, the exit interview (*N* = 30), and the overall study population (*N* = 106) were comparable across key demographic parameters, including age, weight, PS requirements, sex, and race and ethnicity, as well as key baseline disease characteristics (Table 1). However, one notable difference was observed in country distribution: >67% of participants in the exit interview group were from the United States compared with approximately 22% in the overall study population. A chi‐square test confirmed that this difference in country representation was statistically significant (*P* value < 0.0001).

**Table 1:** Descriptive characteristics of patients.

Characteristic	Exit interview population		Trial population (*N* = 106)
	Glepaglutide (*N* = 22)	Placebo (*N* = 8)	
Age at screening, years			
Mean (SD)	57 (13)	53 (15)	55 (12)
Median	59	54	56
Min; Max	20; 73	36; 81	20; 82
Age groups, *n* (%)			
≥18 to <65 years	14 (64)	7 (88)	81 (76)
≥65 to <75 years	8 (36)	0	25 (24)
≥75 years	0	1 (12)	4 (4)
Sex, *n* (%)			
Female	11 (50)	3 (38)	57 (55)
Male	11 (50)	5 (62)	49 (46)
Race, *n* (%)			
American Indian or Alaska Native	0	0	0
Asian	0	0	1 (1)
Black/African American	2 (9)	1 (12)	3 (3)
Native Hawaiian/Pacific Islander	0	0	0
White	18 (82)	6 (75)	89 (84)
Unknown	2 (9)	1 (12)	4 (4)
Not allowed to ask as per local regulation	0	0	9 (9)
Ethnicity, *n* (%)			
Hispanic or Latino	0	0	1 (1)
Not Hispanic or Latino	22 (100)	7 (88)	95 (90)
Not allowed to ask as per local regulation		1 (12)	10 (9)
Weekly PS volume requirements, L/week			
Mean (SD)	13 (7)	21 (7)	14 (8)
Median	12	23	13
Min; Max	3; 28	10; 31	3; 31
SBS anatomical classification, *n* (%)			
Group 1 (jejunostomy)	12 (55)	6 (75)	52 (49)
Group 2 (jejuno‐colonic anastomosis)	8 (36)	2 (25)	48 (45)
Group 3 (jejuno‐ileo‐colonic anastomosis)	2 (9)	0	6 (6)
Stoma, *n* (%)			
Yes	10 (46)	2 (25)	58 (55)
No	12 (54)	6 (75)	48 (45)
Country, *n* (%)			
Germany	0	2 (25)	12 (11)
Denmark	1 (5)	0	8 (8)
United Kingdom	6 (27)	1 (12)	9 (9)
United States	15 (68)	5 (63)	23 (22)

Abbreviations: Max, maximum; Min, minimum; PS, parenteral support; SBS, short bowel syndrome.

### Interview results

#### Impact of SBS‐IF on daily life before the clinical trial

All 30 patients described experiencing various ways in which SBS‐IF impacted their daily lives before the study. The physical impact of SBS‐IF was expressed by 23 patients and included sleep, physical activities, and pain. Sleep disruptions from nightly PS administration caused fatigue, with one patient stating they must “set an alarm clock to stop my stoma pouch from bursting” (patient 44001); one patient shared being “up all night long, every couple of hours because I'm pumping all these fluids into my body” (patient 01004). Seven patients experienced restricted physical activities, as frequent bag emptying or infection concerns hindered exercise. Notably, five patients also reported the impact of chronic pain and discomfort on daily life. Most patients (24/30) experienced loss of freedom and spontaneity, with frequent bathroom trips and ostomy management causing social anxiety and difficulties with travel (patient 44001, patient 01003). These restrictions and inconveniences forced patients to plan their lives around PS needs: “whole day revolved around PN [parenteral nutrition]” (patient 44005). Some patients avoided eating or drinking outside the home because of challenges of managing outputs in social settings (patient 01004, patient 01012). On the psychosocial aspect, 12 patients reported emotions of anger, frustration, embarrassment, hopelessness, and a longing for a normal life, with one describing living with SBS as “a living hell” and further sharing that “there was just no feeling of hope, of anything getting any better” (patient 01010). The debilitating effects of SBS‐IF also influenced professional and academic paths, with one patient retiring early (patient 01012) and another adjusting their university plans to stay near to medical facilities (patient 44005).

#### Patient‐reported changes to daily life since starting the clinical trial

When asked about changes in their daily lives during the clinical study, 73% (16/22) of patients receiving glepaglutide (OW/TW) reported positive changes in daily life, compared with 25% (2/8) receiving PBO. Several patients (8/16 [50%] glepaglutide, 1/2 [50%] PBO) experienced improvements in physical health, citing reduced joint pain and fatigue, and increased energy and appetite (eg, “I have more energy to go out and do things” [patient 01008, OW]). Additionally, seven patients (6/16 [38%] glepaglutide, 1/2 [50%] PBO) reported improved leisure and social life, enabling them to engage in previously avoided activities; for instance, one patient shared, “I started coaching volleyball, which I couldn't do before” (patient 01001, OW). Four patients (4/16 [25%]), all receiving glepaglutide, reported better mental and emotional well‐being, expressing feelings of happiness and optimism due to better PS management and reduced sleep disruptions: “I feel better having an extra night of much less interrupted sleep” (patient 44005, TW). Another four patients (4/16 [25%]) receiving glepaglutide but none in the PBO group experienced increased autonomy, which allowed for more flexibility in daily activities and travel. One patient stated, “I can spend weekends with my girlfriend without worrying about PN” (patient 44005, TW). Two patients (2/16 [13%]) receiving glepaglutide reported improvements in ability to perform daily activities, as exemplified by one patient saying, “I'm able to do more tasks around the house” (patient 01012, TW).

#### Patient‐reported overall change in PS use since starting the clinical trial

A total of 26 patients (20 receiving glepaglutide and six receiving PBO) reported reduced PS volume, which represents 91% (20/22) of the total patients receiving glepaglutide, compared with 75% (6/8) receiving PBO. In line with the predefined decision tree outlined in the interview manual, only these patients were asked to rate their overall status while receiving PS by rating a 7‐point Likert scale. Among them, 21 patients (18/20 [90%] glepaglutide, 3/6 [50%] PBO) reported a change in overall status, whereas five patients (2/20 [10%] glepaglutide, 3/6 [50%] PBO) noted no change. Seven patients, all receiving glepaglutide, rated their status as “very much improved,” and six patients, also all receiving glepaglutide, rated their status as “much improved.” Reasons for their ratings (Table 2) included positive changes such as reduced PS volume and time spent (patient 01002, TW and patient 01013, OW), leading to improved QoL (patient 44006, TW) and greater flexibility; as one patient expressed, “I'm not tied down to a bag for 10 h that day” (patient 01012, TW). Physical benefits such as improved hydration (patient 44002, OW), better sleep (patient 44005, TW), and weight gain from a varied diet (patient 01005, OW) were also highlighted.

**Table 2:** Reasons identified for change in patient's overall status after PS reduction.

Overall change level	Reason for response	Excerpts
Overall status was very much improved (TW = 3 and OW = 4)	‐ Less time spent receiving PS^a^ ‐ No more PS ‐ Less PS volume ‐ Less bathroom dependence ‐ More freedom ‐ Improved QoL	*Because now I don't have to… I don't have the IV fluid every, you know, I don't have to do that every day* (patient 01002, TW).
Overall status was much improved (TW = 3; OW = 3; and PBO = 1)	‐ More freedom^a^ ‐ Improved state of mind ‐ Better sleep ‐ Improved QoL ‐ Less PS volume ‐ Less time receiving PS ‐ Improved hydration ‐ Increased weight ‐ Allowed increased variety of food ‐ Eat more	*Improved flexibility in my daily life, allowing me to‐ giving me, more freedom to do what I want. Better sleep and just general improvement in state of mind, really* (patient 44005, TW).
Overall status was minimally improved (OW = 3 and PBO = 2)	‐ Bag is lighter to carry^a^ ‐ Less PS volume ‐ Less time receiving PS ‐ Life has not changed ‐ Improved hydration ‐ Improved state of mind ‐ Improved QoL	*Yeah, I went from a [inaudible] milliliter TPN bag three times a week, down to 2400. The weight of that bag, just losing that 600 milliliters, was a mountain… it was a godsend because that bag is so heavy. Yeah, it lessened* (patient 01010, OW).
Overall status did not change (OW = 2 and PBO = 3)	‐ Life has not changed^a^ ‐ Experienced dehydration	*As the TPN has dropped as… let's see… to say, that towards the end of the day that I don't have a bag of TPN and through the next day I feel quite a bit more… dehydrated. If that makes sense, and then it sort of catches up* (patient 44007, PBO).
Overall status was minimally worsened (TW = 1 and OW = 1)	‐ Experienced dehydration^a^ ‐ Less energy	*I just feel a bit dry and therefore sometimes not as energetic, I'm sure because of that. The change in parenteral nutrition* (patient 44003, OW).

Abbreviations: IV, intravenous; OW, once weekly; PBO, placebo; PS, parenteral support; QoL, quality of life; TPN, total parenteral nutriition; TW, twice weekly.

^a^ Reasons that correlate to the excerpts.

Of the five patients (2/20 [10%] glepaglutide, 3/6 [50%] PBO) reporting “no change” in their overall status, three patients (1/5 [20%] glepaglutide, 2/5 [40%] PBO) provided reasons. One stated “everything is still the same” (patient 01003, PBO), another mentioned “still on TPN [total parenteral nutrition] 12 h a day” (patient 01015, OW), and a third reported feeling dehydrated on days without PS (patient 44007, PBO).

Two patients (2/20 [10%] glepaglutide) felt their status had “minimally worsened” because of reduced PS. One stated, “I would say minimally worsened because I was getting dry” (patient 44001, TW), and the other reported feeling a bit drier, which affected their energy (patient 44003, OW).

#### Patient‐reported meaningful change in PS volume

For this question, only the 21 patients reporting a change in overall status were asked if “the change in weekly parenteral support need was meaningful.” This was guided by the interview manual's predefined decision‐tree logic framework, which aimed to assess patient‐perceived benefit in the context of both perceived clinical change and subjective well‐being. This required both a PS reduction and improved overall health status. As a result, the seven patients reporting PS reduction without concurrent improvement in overall health status were excluded to adhere to the predefined criteria. This approach was intended to explore patient perspectives on meaningful benefit and to maintain internal consistency across interview responses.

The 21 patients reporting a change in overall health status were then asked whether “the change in weekly parenteral support need was meaningful.” A total of 19 patients (17/18 [94%] receiving glepaglutide and 2/3 [67%] receiving PBO) found the PS reduction meaningful. Patients reported increased autonomy and improved physical and emotional well‐being. Eight patients (8/17 [47%] glepaglutide) described regaining a sense of control and normalcy over their daily lives. Seven patients (6/17 [35%] glepaglutide, 1/2 [50%] PBO) noted improved physical well‐being such as weight gain, better sleep (patient 01007, TW), and improved nutrient absorption (patient 01005, OW). One patient emphasized the impact of even a small reduction in PS time: “two hours might not seem like a whole lot to everybody, but that's two hours every day, seven days a week” (patient 01004, TW). Others appreciated reduced worry about running out of fluids and avoiding the embarrassment of visible intravenous lines “hanging out of you” (patient 01008, OW).

The two patients (1/1 [100%] glepaglutide, 1/1 [100%] PBO) who found the change was not meaningful explained their reasons. One patient noted that despite the reduction in PS volume, “it's still the same amount of time” (patient 01015, OW) required for administration. Another patient shared that using less PS did not change their routine: “Twelve hours [is] still needed. The five days a week [is] still needed and all the negative things of having to carry that thing around, having to have the supplies every week to do the peripheral nutrition, you know none of that changed” (patient 01016, PBO).

#### Patient‐reported meaningful change in physical and emotional symptoms related to SBS

This global topic of discussion aimed to identify and understand patients' appraisal of meaningful changes in the different QoL domains including (1) sleep related to SBS, (2) ability to do what one wants to do, (3) exhaustion or tiredness, (4) mood related to SBS, and (5) stress or anxiety. Using the 7‐point Likert rating scale, patients were asked to rate their experienced change as either “very much improved,” “much improved,” “minimally improved,” “not changed,” “minimally worsened” “much worsened,” or “very much worsened” in these domains.

##### Patient‐reported meaningful change in exhaustion or tiredness

**Figure 2:**
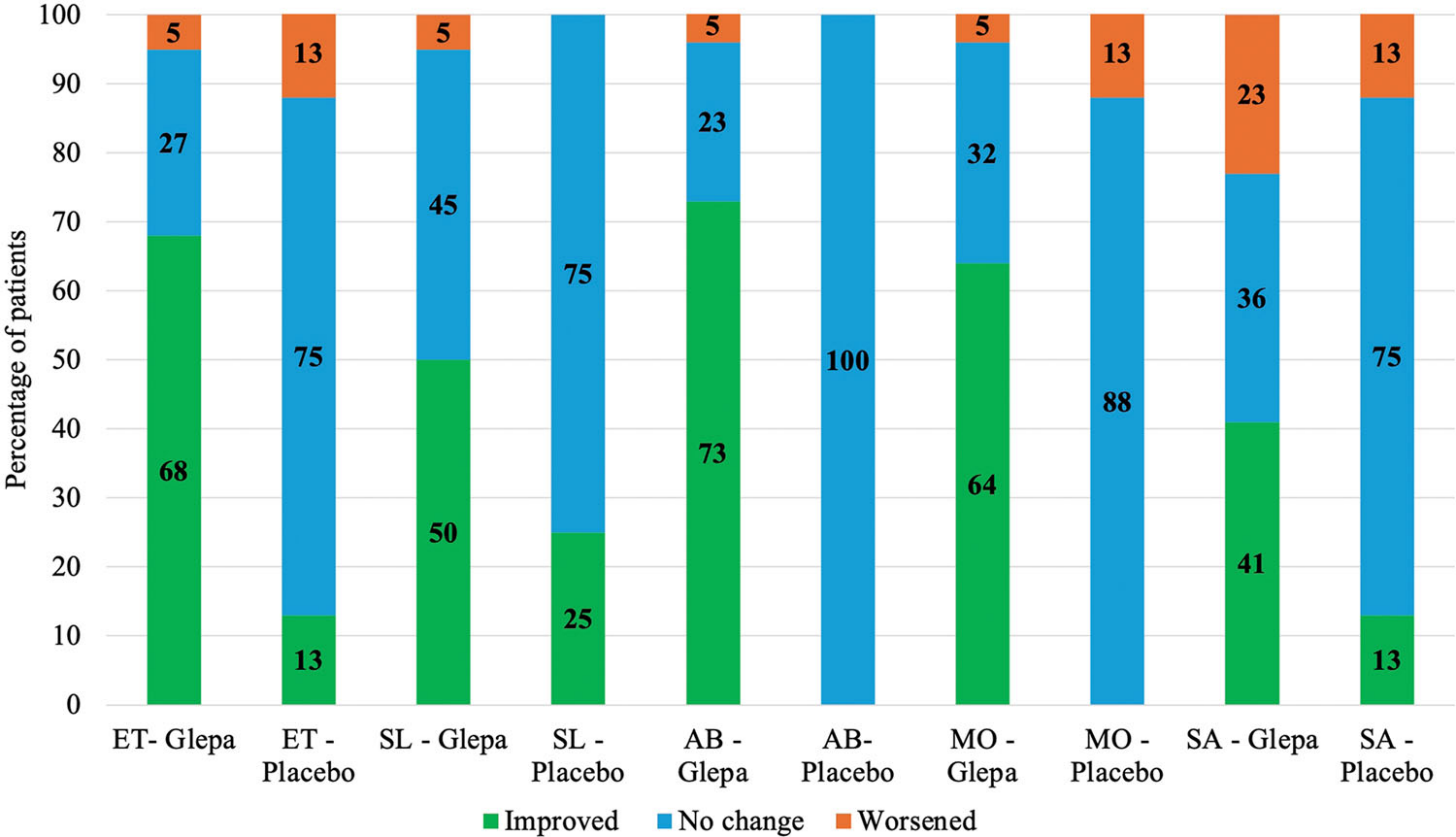
Patient‐reported change in exhaustion or tiredness, sleep, ability to do what one wants to do, mood, and stress or anxiety among the interview population (*N* = 30) (Glepa, *N* = 22; placebo, *N* = 8). “Very much improved,” “Much improved,” and “Minimally improved” were combined to represent the category “Improved.” Similarly, “Minimally worsened,” “Much worsened,” and “Very much worsened” were combined to represent the category “Worsened.” The outcome “Did not change” was kept as a standalone category. AB, ability to do what one wants to do; ET, exhaustion or tiredness; Glepa, glepaglutide; MO, mood; SA, stress or anxiety; SL, sleep.

Of the 30 patients, 16 patients (15/22 [68%] glepaglutide, 1/8 [13%] PBO) reported an improvement in exhaustion or tiredness, whereas two patients (1/22 [5%] glepaglutide, 1/8 [13%] PBO) reported a worsening status (Figure 2). Most of the patients experiencing this improvement attributed the meaningful change to increased vitality, return to normalcy, and increased engagement in physical activities (Table 3). Examples included living a more active lifestyle (patient 01012, TW), completing daily tasks (patient 01008, OW), and feeling less exhausted during light tasks (patient 01011, OW). Additionally, two patients felt that the reduction in exhaustion or tiredness allowed them to return to a more normal life.

**Table 3:** Patient‐reported meaningful change excerpts in exhaustion or tiredness, sleep, ability to do what one wants to do, mood, and stress or anxiety.

Domain	Themes	Excerpts
Exhaustion or tiredness	Improved vitality	*Yes, the exhaustion is less than it was. The TPN corrected it, but only for the day. And then it started all over again, you see. So far, while I'm still having that exhausting… it's when I exert myself, does that make sense? Before it was, I could be sitting and reading and get exhausted* (patient 01011, OW).
	Return to normalcy	*It allows me to, you know, get on with my life, not being constantly in the bed. And just, you know, just being able to move on with my life* (patient 01002, TW).
Sleep	More awake/alert during the day	*It's mostly that, I think. The not having such broken sleep, I think actually getting through the sleep cycle instead of being always interrupted, it's leaving me a bit more alert for the day* (patient 44007, PBO).
	Hope of better future	*It's going to be awesome… speaking for myself, you'd only lay for two hours and sleep, you have to get up. You never get into REM. So, I haven't had a dream in years* (patient 01004, TW).
Ability to do what one wants to do	Able to do more daily and social activities	*It feels good to… My house is a mess. So being able to do little things to help my husband clean and… That improves my life because it makes me feel better and it takes some of the burden off him* (patient 01015, OW).
	Regaining normal life	*Again, it's like that freedom, I feel like I can actually start to work [inaudible] to doing things and doing some things again* (patient 01008, OW).
Mood	Improved functionality	*And just be able to function more as I used to before health issues took over my life. And that I can respond to my children, and love and do these things that… You know live the way that I want to live* (patient 01001, OW).
	Improved psychosocial state	*Oh, it makes it more enjoyable to be around me. Basically, you know if you're in a happier place and things seem happier* (patient 01015, OW).
Stress or anxiety	Positive emotions	*Yeah. You know, I've always been a very relaxed person and I can relax more now. You know. Worrying about, you know. What's happening now, and whatever* (patient 01013, OW).
	Any improvement is meaningful	*I would definitely say that any little improvement in my stress level is going to be a meaningful improvement for not just me, but for my wife and family* (patient 01009, OW).

Abbreviations: OW, once weekly; PBO, placebo; REM, rapid eye movement; TPN, total parenteral nutrition; TW, twice weekly.

##### Patient‐reported meaningful change in sleep

Of the 30 patients, 13 patients (11/22 [50%] glepaglutide, 2/8 [25%] PBO) reported an improvement in their sleep, whereas one patient (1/22 [5%] glepaglutide) noted a minimal worsening (Figure 2). All 14 patients who reported a change in sleep described the change as meaningful. Five patients (4/14 [29%] glepaglutide, 1/14 [7%] PBO) reported less interrupted sleep and a more regular sleep cycle contributed to them feeling more awake and alert during the day (Table 3). One patient explained, “I'm better rested*…* not giving up so much*…* I just keep myself going. I would say sometimes I'd just have to give myself a little push and off I go” (patient 01007, TW). Improved rest allowed patients to return to normalcy and better reintegration into family life and society (“function in society” [patient 01014, OW]). For others, better sleep fostered a more active lifestyle and a renewed hope for a brighter future, which previously seemed implausible. The one patient who reported “minimally worsened” sleep attributed this to pump malfunctioning at night “because there is an air in the line,” which prevented them from getting a full 8 h of sleep despite reducing their hours of PS (patient 01006, OW).

##### Patient‐reported meaningful change in ability to do what they want to do

Sixteen patients (16/22 [73%]), all receiving glepaglutide, reported an improvement in their ability to do what they want to do, whereas one patient receiving glepaglutide noted a minimal worsening (Figure 2). Comparatively, all eight patients (100%) receiving PBO reported no change. Change was attributed to several factors (Table 3), with seven patients reporting participation in daily and social activities such as running errands (patient 01013, OW), assisting with household chores (patient 01012, TW), attending family and social events and being able to “be out in society fulfilling my duties” (patient 01014, OW), and returning to work (patient 01008, OW). Patients also expressed how these changes helped them regain control of their lives, with one patient relaying, “Just in that my life has returned more to normalcy. So, before I had the SBS condition, my life is more like what it was before I had the SBS” (patient 01009, OW). One patient shared how their improved ability fostered a renewed sense of drive and purpose (“more drive to persevere, going out”), particularly overcoming past suicidal thoughts (patient 01010, OW). Some patients reported positive emotional well‐being such as newfound hope and feeling a sense of relief (patient 01015, OW) and feeling worry‐free (patient 01011, OW). In contrast, the one patient who noted a minimal worsening felt demotivated and adopted an “I can't be bothered” attitude (patient 44001, TW), which they found meaningful, as fatigue and lack of motivation disrupted their ability to engage in valued activities such as working on cars, something they previously enjoyed and felt strongly connected to.

##### Patient‐reported meaningful change in mood related to SBS

Fourteen patients (14/22 [64%]), all receiving glepaglutide, reported an improvement in their mood, whereas two patients (1/22 [5%] glepaglutide, 1/8 [13%] PBO) experienced worsening (Figure 2). The most cited reason for improvement was improved functionality, with patients feeling they could resume daily activities and regain independence (Table 3). One patient noted, “I had to rely on my husband to do a lot of this stuff. And now I can either help him or he can help me. Or I can do it myself. If I can do it myself, I'd rather do that” (patient 01012, TW). Additionally, six patients reported a better psychosocial state, a more positive feeling, improvement in relationships, and participation in activities. One patient felt relieved from financial stress because of the reduced costs of PS. However, one patient (patient 44005, TW) who reported a worsening in mood felt demoralized because of fleeting hope for health improvements.

##### Patient‐reported meaningful change in stress or anxiety related to SBS

Ten patients (9/22 [41%] glepaglutide, 1/8 [13%] PBO) reported improvements in stress or anxiety, whereas six patients (5/22 [23%] glepaglutide, 1/8 [13%] PBO) noted minimal worsening (Figure 2). Thirteen patients found the changes meaningful. Two patients attributed this to positive emotions (Table 3), such as relief from worries about dragging the pole around (patient 01013, OW) and reflection on their experience with greater hope and less stress (patient 01014, OW). Two patients (OW) emphasized that any improvement, regardless of size, was meaningful: “Anything which helps me and is better is meaningful, of course. Can I quantify it? Can I explain it? No” (patient 44008, OW). One patient attributed the change to having good medical care and support during the trial (patient 01015, OW). Of the six patients reporting on minimal worsening, four attributed it to trial requirements and procedures rather than treatment.

## DISCUSSION

The exit interview substudy was part of the interventional phase 3 EASE SBS‐1 trial (ClinicalTrials.gov, NCT03690206). The aim of the exit interviews was to establish patients' lived experiences and to assess the impact of glepaglutide treatment in the exit interview substudy. This included explicating (1) subjective individual patient assessment of meaningful changes in PS need and (2) meaningfulness of change in physical and emotional symptoms that are most relevant. Patient exit interviews yielded important results to support findings from the main trial.^13^

When asked to describe the impact of SBS on their lives before enrolling in the clinical trial, patients described the physical and psychosocial challenges of living with SBS‐IF, highlighting the restrictions on their ability to perform activities of daily living. Many experienced physical limitations, including disrupted sleep and dietary restrictions. These limitations often led to feelings of anger, frustration, embarrassment, and hopelessness regarding their condition and the concomitant restrictions. Patient narratives reflected a pervasive sense of loss of freedom and spontaneity, with many needing to plan their lives around HPS and the demands of SBS.^16^, ^17^ The literature is unequivocal about the significant life changes imposed by SBS and the varying degrees of PS needs,^10^, ^11^, ^18^ a burden confirmed in the exit interviews, which also affected families and care providers.

Most patients receiving glepaglutide experienced improvements across various aspects of life. However, because of the unequal distribution of patients between the glepaglutide and PBO groups, caution is required when comparing these results with those of the PBO group. Most patients, including some in the PBO group, described a reduction in the disease burden associated with SBS‐IF. Similarly, teduglutide, a short‐acting GLP‐2 analogue, had a positive impact on SBS‐related QoL by lessening the required volume of PS.^18^, ^19^ For some patients, weight gain and an increased appetite resulted in a sense of increased energy and the ability to engage in previously difficult activities. The reduction in PS volume came along with better physical and psychological well‐being, providing patients with more freedom and autonomy to participate in social activities. This fostered a sense of optimism and excitement about their futures, as patients felt renewed hope for reintegrating into “normal society.”

Clinical outcome end points from the pivotal EASE SBS‐1 trial showed that a 24‐week regimen of TW glepaglutide effectively reduced or even eliminated the need for PS in patients with SBS‐IF, whereas nonstatistically significant reductions in weekly PS volume occurred with OW dosing compared with placebo.^13^ The reductions in PS volume are induced by increases in intestinal absorption, which results in less stomal output and uncontrollable diarrhea, and in reduced PS infusion time. This also translates to more days off PS or even the possibility of achieving enteral autonomy. Patients in the EASE SBS‐1 study rated their perception of change from baseline after the 24‐week intervention by using the PGIC scale. Patients receiving glepaglutide, especially those in the TW‐dose arm, were significantly more likely to rate their perception of change as “much or very much improved” compared with the PBO arm. Qualitative data from the exit interviews further support these quantitative findings. Many patients in the glepaglutide group reported meaningful reductions in PS use and expressed satisfaction with the associated improvements in daily life. They were optimistic about further improvements in functioning and a reduction in PS usage. Consistent with previous studies, patients with SBS‐IF describe their ideal treatment as one that reduces PS usage for a few hours or nights, thereby restoring their sense of freedom, autonomy, and normalcy.^11^

The interviews highlighted a complex relationship between PS reduction and patient‐reported symptom burden, such as less output from an uncontrollable stoma or diarrhea, which can be influenced by what an individual patient considers “meaningful change.” As a result, patients reported diverse responses in “improvement” or “worsening” of symptoms they considered meaningful. For example, one patient receiving glepaglutide reported a reduction in PS volume but subsequently did not experience any change in exhaustion or tiredness, sleep, mood, and stress or anxiety. This variability underscores two key issues. First, what patients consider meaningful change differs widely, and second, the concept of meaningfulness itself varies among patients. Although reducing reliance on PS might seem beneficial, its impact on QoL is complex because disease burden and meaningful change are highly individualized. Given the heterogeneity of SBS‐IF, caution is required when interpreting meaningful change and what is important for SBS‐IF patients at a population level. The qualitative interviews, however, offer valuable insights into this variability, helping to contextualize the quantitative data and providing a deeper understanding of what matters most to patients.

It is important to recognize some limitations when evaluating the exit interview trial results. First, SBS‐IF is a rare condition with a large patient heterogeneity, resulting in small sample sizes that may skew the results toward a more represented group, thus reducing overall generalizability, thereby affecting the generalizability of the findings. Although the participants in the exit interviews were largely comparable to the overall EASE SBS‐1 study population across demographic and clinical variables, the chi‐square analysis revealed a statistically significant difference in the “country” variable. Such selection bias may limit the diversity of perspectives and reduce the study's ability to capture regional variations, as participants from certain areas may be overrepresented or underrepresented. Finally, there was an imbalance in participant numbers between the glepaglutide and PBO groups in the exit interviews. Despite these limitations, the results offer valuable insights into patients' experiences with treatment impacts. The exit interviews also allowed for rich exploration of qualitative themes, offering a deeper understanding of how treatment is perceived and what constitutes meaningful change from the patients' perspectives.

In conclusion, consistent with the pivotal trial, the exit interviews highlighted that treatment with glepaglutide provided benefits in both disease burden and QoL. Patients expressed satisfaction with these changes, although the interviews also highlighted variability in what individuals perceived as meaningful improvements. Overall, the results provide valuable insights into the patient experience and demonstrate the potential for glepaglutide to address challenges on patients' well‐being across multiple domains, thus informing future patient‐centered SBS‐IF care strategies.

## AUTHOR CONTRIBUTIONS

David F. Mercer, Constance Mubekapi‐Musadaidzwa, and Palle Bekker Jeppesen led the investigation of the research, with Bitten Kloster, Mark Berner‐Hansen, and Anna Rydén providing support. Constance Mubekapi‐Musadaidzwa, Bitten Kloster, Mark Berner‐Hansen, and Palle Bekker Jeppesen led data visualization, with supporting contributions from David F. Mercer and Anna Rydén. Constance Mubekapi‐Musadaidzwa and Bitten Kloster led the writing of the manuscript, with supporting contributions from David F. Mercer, Mark Berner‐Hansen, Anna Rydén, and Palle Bekker Jeppesen. All authors contributed to the review and editing of the manuscript, with equal contributions from David F. Mercer, Constance Mubekapi‐Musadaidzwa, Bitten Kloster, Mark Berner‐Hansen, and Palle Bekker Jeppesen. All authors critically revised the manuscript, agree to be fully accountable for ensuring the integrity and accuracy of the work, and read and approved the final manuscript.

## CONFLICT OF INTEREST STATEMENT

David F. Mercer received consultant fees from Zealand Pharma and Takeda and is a primary investigator in clinical trials sponsored by Zealand Pharma, Ironwood, and Takeda. Bitten Kloster and Mark Berner‐Hansen are employees of Zealand Pharma, who sponsored the clinical trial. Palle Bekker Jeppesen received research support from Albumedix A/S, ArTara Therapeutics, Bainan Biotech, Baxter, Coloplast A/S, Ferring Pharmaceuticals, Fresenius Kabi, GLyPharma Therapeutic, Hanmi Pharmaceuticals, Ironwood, Naia Pharmaceuticals, Novo Nordisk Foundation, NPS Pharmaceuticals, Protara Therapeutics, Shire, Takeda, Therachon, and Zealand Pharma. The remaining authors declare no conflicts of interest.
